# Supplementary material for: Acute Kidney Failure among Brazilian Agricultural Workers: A Death-Certificate Case-Control Study
Source: Int J Environ Res Public Health. 2022 May 27;19(11):6519. doi: 10.3390/ijerph19116519 (PMC9179952; doi:10.3390/ijerph19116519)
Supplement: Supplementary file 1 [file ijerph-19-06519-s001.zip › ijerph-1629970-supplementary.pdf]

Supplementary Table S1 - AKF mortality among agricultural workers according to the 10-year birth cohort.

	**Cases**<br>**N (%)**	**Controls**<br>**N (%)**	**OR* (95% CI)**	**OR** (95% CI)**
<u>**Cohort**</u>				
**1920-1929**				
Non-Agricultural workers	1,075 (76.8)	259,076 (80.6)	1.00	1.00
Agricultural workers	325 (23.2)	62,249 (19.4)	1.26 (1.11-1.43)	1.29 (1.12-1.48)
**1930-1939**				
Non-Agricultural workers	1,071 (76.8)	327,654 (81.6)	1.00	1.00
Agricultural workers	324 (23.2)	73,941 (18.4)	1.34 (1.18-1.52)	1.37 (1.20-1.57)
**1940-1949**				
Non-Agricultural workers	689 (80.4)	267,119 (84.0)	1.00	1.00
Agricultural workers	168 (19.6)	51,008 (16.0)	1.28 (1.08 -1.51)	1.28 (1.07 - 1.55)
**1950-1959**				
Non-Agricultural workers	467 (87.1)	203,297 (86.0)	1.00	1.00
Agricultural workers	69 (12.9)	32,959 (14.0)	0.91 (0.71 -1.17)	0.94 (0.72 - 1.24)
**1960-1969**				
Non-Agricultural workers	212 (85.5)	132,584 (87.6)	1.00	1.00
Agricultural workers	36 (14.5)	18,759 (12.4)	1.20 (0.84 -1.71)	1.41 (0.96 - 2.07)
**1970-1979**				
Non-Agricultural workers	105 (85.4)	82,719 (89.6)	1.00	1.00
Agricultural workers	18 (14.6)	9,596 (10.4)	1.48 (0.90 -2.44)	1.73 (0.99 - 3.04)
**1980-1994**				
Non-Agricultural workers	62 (89.9)	78,242 (92.5)	1.00	1.00
Agricultural workers	7 (10.1)	6,378 (7.5)	1.39 (0.63-3.03)	1.71 (0.77-3.79)

*Crude odds ratio; **Odds ratio adjusted by all other variables
